# A Rare Case of Duodenal Melanosis: Case Report

**DOI:** 10.7759/cureus.10475

**Published:** 2020-09-15

**Authors:** Navpreet K Rana, Umair Minhas, Thomas Mahl

**Affiliations:** 1 Internal Medicine, University at Buffalo, Buffalo, USA; 2 Gastroenterology and Hepatology, Division of Gastroenterology, Hepatology and Nutrition, University at Buffalo, Buffalo, USA

**Keywords:** pseudomelanosis, duodenal melanosis, upper endoscopy, small bowel

## Abstract

Pseudomelanosis (PM) is a rare condition of unknown etiology and pathogenesis, described as speckled black pigmentation of intestinal mucosa. It is usually discovered as an incidental finding during endoscopy. Although, etiology of PM is unclear, it has been associated with different medications and systemic diseases such as chronic renal disease and diabetes mellitus. In this report, we describe a case of a 72-year-old male with multiple co-morbidities who presented with epigastric pain, nausea and hematemesis. Subsequently, upper endoscopy performed revealed intestinal PM with no active bleeding. Although considered a benign condition, knowing the existence of PM is important to exclude other serious conditions with similar endoscopic findings.

## Introduction

Pseudomelanonis (PM) of the gastrointestinal tract is a benign condition that is characterized by the presence of brown or black dark spots in the gastrointestinal mucosa during endoscopy. It is usually reported as an incidental finding on upper endoscopy, mainly found in the duodenum, but has also been reported in the stomach and jejunum [1,2]. It was first described by Bisordi and Kleinman in 1976, as “melanosis duodeni”, or black pigmentation of the duodenal mucosa on endoscopy [3]. Castellano et al. showed histologically, these black pigments are located in the lysosomes of macrophages in the lamina propria [4]. Electron microscopy and histochemical studies have shown, PM pigments result from the accumulation of compounds such as iron sulfide, ferrum sulfate, ceroid, hemosiderin or lipomelanin within the lysosomes of macrophages in lamina propria [5-7]. This condition is defined as “pseudomelanosis” because unlike the melanosis coli, which is associated with the overuse of anthraquinone containing laxatives, PM does not contain lipofuscin [7,8]. The underlying cause of PM is not clear; however, multiple case reports have been published, reporting its association with certain medications such as hydralazine, ferrous sulfate, furosemide, or propranolol or with systemic diseases such as hypertension, chronic renal disease or diabetes mellitus [6,9-11]. Although it is a benign condition and produces no symptoms, it is usually diagnosed in patients who present with other symptoms that warranting endoscopic evaluation [12].

## Case presentation

A 72-year-old male with a medical history of peripheral arterial disease (PAD), mesenteric ischemia status post superior mesenteric and celiac artery stents, gastroesophageal reflux disease, hypertension, diabetes, chronic kidney disease, hypothyroidism and chronic obstructive pulmonary disease, presented with one-day history of epigastric pain, nausea and hematemesis. The patient denied any prior episodes of melena or bright red blood per rectum and reported no personal history of rectal or colon cancer. He also denied the use of nonsteroidal anti-inflammatory drugs, iron supplements or bismuth subsalicylate. He denied the use of alcohol, tobacco or recreational drugs. His medications included aspirin, clopidogrel, amlodipine, hydralazine, hydrochlorothiazide, atorvastatin, levothyroxine, furosemide and baclofen.

**Figure 1:**
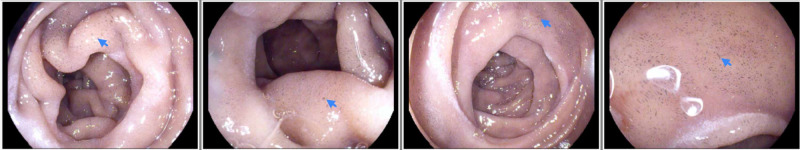
Dark pigmentation of the duodenal mucosa noted on upper endoscopy. Pigments marked with blue arrows.

**Figure 2:**
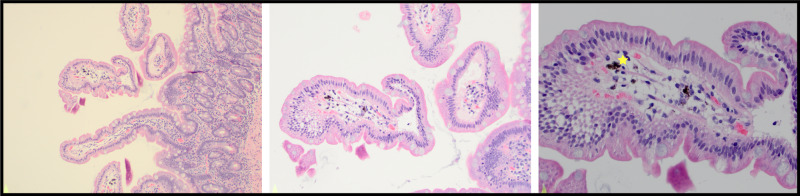
Histochemical study illustrating pigment in the duodenal mucosa, marked with yellow star.

On admission, the patient was hemodynamically stable, his hemoglobin (HgB) was 9.2 with mean corpuscular volume (MCV) of 93.2. Eight months ago, his HgB was 11.4 with MCV of 89.9. Iron studies, level of folate and vitamin B12 were all within normal limits. Digital rectal exam showed dark stool. His aspirin and clopidogrel were held and he was started on intravenous pantoprazole 40 mg twice daily. Patient underwent upper endoscopy to identify the source of his gastrointestinal bleeding. It was unremarkable with no blood seen or identifiable source of bleeding. The esophagus, gastric mucosa and gastroesophageal junction were all normal in appearance. Dark pigmentation of the duodenal mucosa was noted and subsequently biopsied (Figure 1). Evaluation of the biopsied sample was consistent with intestinal PM (Figure 2) with no evidence of dysplasia or malignancy. Patient’s initial complains of nausea and hematemesis resolved without any additional intervention. He was transitioned to oral proton-pump inhibitor. Given his history of PAD, he was continued on aspirin, but his clopidogrel was discontinued upon discharge.

## Discussion

Exact pathophysiology of PM is unclear, but medications and certain disorders have been implicated. In a study by Giusto and Jakate in 2007, the medical history of 17 patients with histological findings of PM was evaluated. Of these 17 patients, 88% had history of hypertension, 59% had end-stage renal disease and 35% had diabetes mellitus [9]. Medications that were common among these patients were iron supplements and different classes of anti-hypertensives such as hydrochlorothiazide, atenolol, lisinopril/quinapril and irbesartan [9]. Other case reports have described PM as an incidental finding in patients presenting with gastrointestinal bleeding, similar to our patient [13,14]. The mechanism by which these diseases and medications contribute to PM is not known. Current theories have speculated that accumulation of iron sulfide in macrophages is likely secondary to impaired transport of iron in the proximal duodenum, which is the most common site of PM, coupled with sulfur from anti-hypertensive medications [15]. Relationship between impaired iron absorption and PM could also explain the finding of PM in patients with chronic kidney disease, since these patients are commonly on iron supplements. This theory, however, does not explain the findings of PM in other areas of the GI tract including the stomach and the jejunum which are not involved in iron absorption. Our patient had several of these risk factors, including his medications and his history of hypertension, diabetes and chronic kidney disease which could have contributed to the finding of PM.

Although PM can be identified endoscopically, biopsy of the gastrointestinal mucosa is imperative to distinguish this benign finding from other conditions such as metastatic malignant melanoma, brown bowel syndrome, hemosiderosis and hemochromatosis [16-18]. Kaplan et al. described a case of a metastatic malignant melanoma associated with “panenteric melanosis”, described as pigment deposition throughout the GI tract [17]. Biopsy of these pigmented lesions and subsequent staining with Fontana-Masson stains confirmed the presence of melanin deposits. Since Fontana-Masson stain is not specific for melanin, other immunohistochemical stains can also be used to distinguish PM from malignant melanoma. Histiocytes in PM stain positive for CD168 and CD68, whereas malignant melanoma cells may stain positive for S100 or tyrosinase among other agents [16]. Similarly, iron deposits in hemosiderosis stain strongly with Prussian blue iron stain, whereas PM displays partial or weak staining for iron [19]. In some cases, PM cannot be stained with Prussian blue stain, and this has been attributed to iron existing in the form of iron sulfide in PM, instead of iron oxide [16,20].

## Conclusions

In summary, PM is clinically a rare and benign condition that is often found incidentally during upper endoscopy, and is not associated with any particular symptoms. Histologically, it is identified as the accumulation of pigmented granules in macrophages located in the lamina propria. It is important to be aware of this finding and be able to differentiate it from other conditions, which present with similar findings.

## References

[1] Rustagi T, Mansoor MS, Gibson JA, Kapadia CR (2015). Pseudomelanosis of stomach, duodenum, and jejunum. J Clin Gastroenterol.

[2] Weinstock LB, Katzman D, Wang HL (2003). Pseudomelanosis of stomach, duodenum, and jejunum. Gastrointest Endosc.

[3] Bisordi WM, Kleinman MS (1976). Melanosis duodeni. Gastrointest Endosc.

[4] Castellano G, Canga F, Lopez I, Colina F, Gutierrez J, Costa R, Solis-Herruzo JA (1988). Pseudomelanosis of the duodenum. Endoscopic, histologic, and ultrastructural study of a case. J Clin Gastroenterol.

[5] Ghadially FN, Walley VM (1995). Pigments of the gastrointestinal tract: a comparison of light microscopic and electron microscopic findings. Ultrastruct Pathol.

[6] West B (1988). Pseudomelanosis duodeni. J Clin Gastroenterol.

[7] Sharp JR, Insalaco SJ, Johnson LF (1980). "Melanosis" of the duodenum associated with a gastric ulcer and folic acid deficiency. Gastroenterology.

[8] Moore JD, Baichi M, Toledo R, Sitrin M (2007). Pseudomelanosis of jejunum and ileum. Gastrointest Endosc.

[9] Giusto D, Jakate S (2008). Pseudomelanosis duodeni: associated with multiple clinical conditions and unpredictable iron stainability - a case series. Endoscopy.

[10] Thure Caire M, Kalan S, Brady P, Gill J (2014). Pseudomelanosis of the stomach and duodenum: an uncommon endoscopic finding. Endosc Int Open.

[11] Nakanishi Y, Jetly-Shridhar R, De Felice K (2019). A case of pseudomelanosis duodeni: striking endoscopic features with subtle but characteristic pathologic findings. Int J Surg Pathol.

[12] Cheng CL, Chen PC, Chen TC (2000). Pseudomelanosis duodeni: case report. Chang Gung Med J.

[13] Sunkara T, Caughey ME, Gaduputi V (2018). Rare finding of concomitant pseudomelanosis of stomach and duodenum: case report and literature review. Gastroenterol Hepatol Bed Bench.

[14] Zakaria A, Abdu B, Al Share B, Manabat M, Ngo K (2018). Pseudomelanosis intestini "from pylorus to jejunum:" a rare endoscopic finding in a patient with GI bleeding. J Family Med Prim Care.

[15] Almeida N, Figueiredo P, Lopes S (2009). Small bowel pseudomelanosis and oral iron therapy. Dig Endosc.

[16] Abumoawad A, Venu M, Huang L (2015). Pseudomelanosis duodeni: a short review. Am J Digest Dis.

[17] Kaplan MR, Knittel DR, Lawson P, Schafer TW (2005). Panenteric melanosis: an ominous endoscopic finding. Gastrointest Endosc.

[18] Hong SS, Min YI, Yang SK (2006). Melanosis of the colon and terminal ileum associated with primary malignant melanoma of the anorectum. Gastrointest Endosc.

[19] Pounder DJ, Ghadially FN, Mukherjee TM. (1982). Ultrastructure and electron-probe x-ray analysis of the pigment in melanosis duodeni. J Submicrosc Cytol.

[20] Fernando SS (1990). Pseudomelanosis duodeni: a case report with electron-probe X-ray analysis. Pathology.

